# Juxtacrine Activity of Estrogen Receptor α in Uterine Stromal Cells is Necessary for Estrogen-Induced Epithelial Cell Proliferation

**DOI:** 10.1038/s41598-017-07728-1

**Published:** 2017-08-21

**Authors:** Wipawee Winuthayanon, Sydney L. Lierz, Karena C. Delarosa, Skylar R. Sampels, Lauren J. Donoghue, Sylvia C. Hewitt, Kenneth S. Korach

**Affiliations:** 1 School of Molecular Biosciences, Center for Reproductive Biology, College of Veterinary Medicine, Washington State University, Pullman, Washington 99164 United States; 2 Reproductive and Developmental Biology Laboratory, National Institute of Environmental Health Sciences, National Institutes of Health, Department of Health and Human Services, Research Triangle Park, North Carolina, 27709 United States

## Abstract

Aberrant regulation of uterine cell growth can lead to endometrial cancer and infertility. To understand the molecular mechanisms of estrogen-induced uterine cell growth, we removed the estrogen receptor α (*Esr1*) from mouse uterine stromal cells, where the embryo is implanted during pregnancy. Without ESR1 in neighboring stroma cells, epithelial cells that line the inside of the uterus are unable to grow due to a lack of growth factors secreted from adjacent stromal cells. Moreover, loss of stromal ESR1 caused mice to deliver fewer pups due in part due to inability of some embryos to implant in the uterus, indicating that stromal ESR1 is crucial for uterine cell growth and pregnancy.

## Introduction

In female mammals, 17β-estradiol (E_2_), an endogenous estrogen, is primarily produced by the granulosa cells of the ovaries. E_2_ exerts its activity through estrogen receptors α and β (ESR1 and ESR2)^1^. Upon E_2_ binding, ESRs are dimerized, translocated from the cytoplasm into the nucleus, recruited onto targeted DNA sequences, where they initiate or repress transcription of E_2_-target genes in both non-reproductive and reproductive organs^2^. Female reproductive tissues including mammary glands, ovaries, oviducts, and the uterus, express both ESR1 and ESR2^3^. In the uterus, ESR1 is the major subtype and is expressed in all cell layers: epithelia (monolayer of cells lining the uterine lumen), stroma (connective tissue in the endometrial lining between epithelia and myometrium), and myometrium (muscle cell layer).

Estrogens induce cell proliferation and growth in both reproductive and non-reproductive tissues (such as osteoblasts and hepatocytes). It has been shown that E_2_ selectively stimulates proliferation of uterine epithelial cells in adult ovariectomized mice^4–6^. Tissue recombination studies using isolated epithelial and stromal cells from wild-type or *Esr1* ^−/−^ neonatal uterine tissues transplanted under the kidney capsule showed that ESR1 is not required in uterine epithelial cells for their proliferation. We have confirmed this observation using an adult epithelial cell specific knockout mouse model (*Wnt7a* ^Cre/+^; *Esr1* ^f/f^)^7, 8^, with an intact (no tissue disruption and recombination) uterine tissue structure. The results from these two studies support a mechanism in which E_2_ treatment and activation of ESR1 in the stromal cells produces mitogenic factors, including insulin-like growth factor 1 (IGF1)^9, 10^, and transforming growth factor^11^, which stimulate their subsequent signaling cascades in uterine epithelial cells, leading to cell proliferation. However, a functional requirement of stromal ESR1 in the normal uterine environment *in vivo* has not yet been explored.

In addition to cell proliferative events accompanying E_2_ treatment, we also evaluated the role of stromal ESR1 in female reproductive functions in this study. In normal mouse reproduction, the presence of a copulatory plug is observed the morning after mating is designated 0.5 days post coitus (dpc). At 0.5 dpc, the oocytes are fertilized by the sperm and during 3.0 dpc the embryos develop into morulas or blastocysts within the oviducts (known as Fallopian tubes in humans). In rodents, the blastocysts transit the oviduct to the uterus where they implant exclusively onto the anti-mesometrial pole of the uterine wall at approximately 4.0 dpc^12^. Embryo attachment requires secretion of leukemia inhibitory factor (LIF), an implantation facilitating cytokine, from uterine glands located in the anti-mesometrial pole of the uterus^13^. After embryo implantation, the uterine endometrium undergoes a decidual response (called decidualization), in which the stromal cells proliferate and differentiate into decidua^14^. The decidual cells surrounding embryos provide nutrients and support for the developing fetus before the placenta starts to fully function. The placenta forms on the mesometrial pole of the uterus, where the blood vessels are supplied via the uterine broad ligament. These implantation and decidualization processes are orchestrated by ovarian steroid hormones (E_2_ and progesterone; P_4_) through ESR1 and progesterone receptor (PGR)^15, 16^.

We previously showed that female mice with a global deletion of ESR1 (*Esr1* ^−/−^) are infertile, in part due to an implantation defect^15^. Using a female reproductive tract epithelial cell ESR1 null mouse model (*Wnt7a* ^Cre/+^; *Esr1* ^f/f^), our group and others have demonstrated that uterine epithelial ESR1 is crucial for embryo implantation, decidual response, and fertility^8, 17^. From these previous findings, we hypothesized that a lack of stromal ESR1 could lead to aberrant uterine cell proliferation, while not affecting embryo implantation or uterine decidual response. To test our hypothesis, we have generated a mouse model lacking stromal ESR1, specifically at the anti-mesometrial pole of the uterus. Here we report that stromal ESR1 is required for epithelial cell proliferation. Surprisingly, stromal ESR1 in the uterine anti-mesometrium is also crucial for optimal embryo implantation and artificially induced-decidualization.

## Results

### Stromal ESR1 underlying uterine epithelial cells is essential for E_2_-induced proliferative response

**Figure 1:**
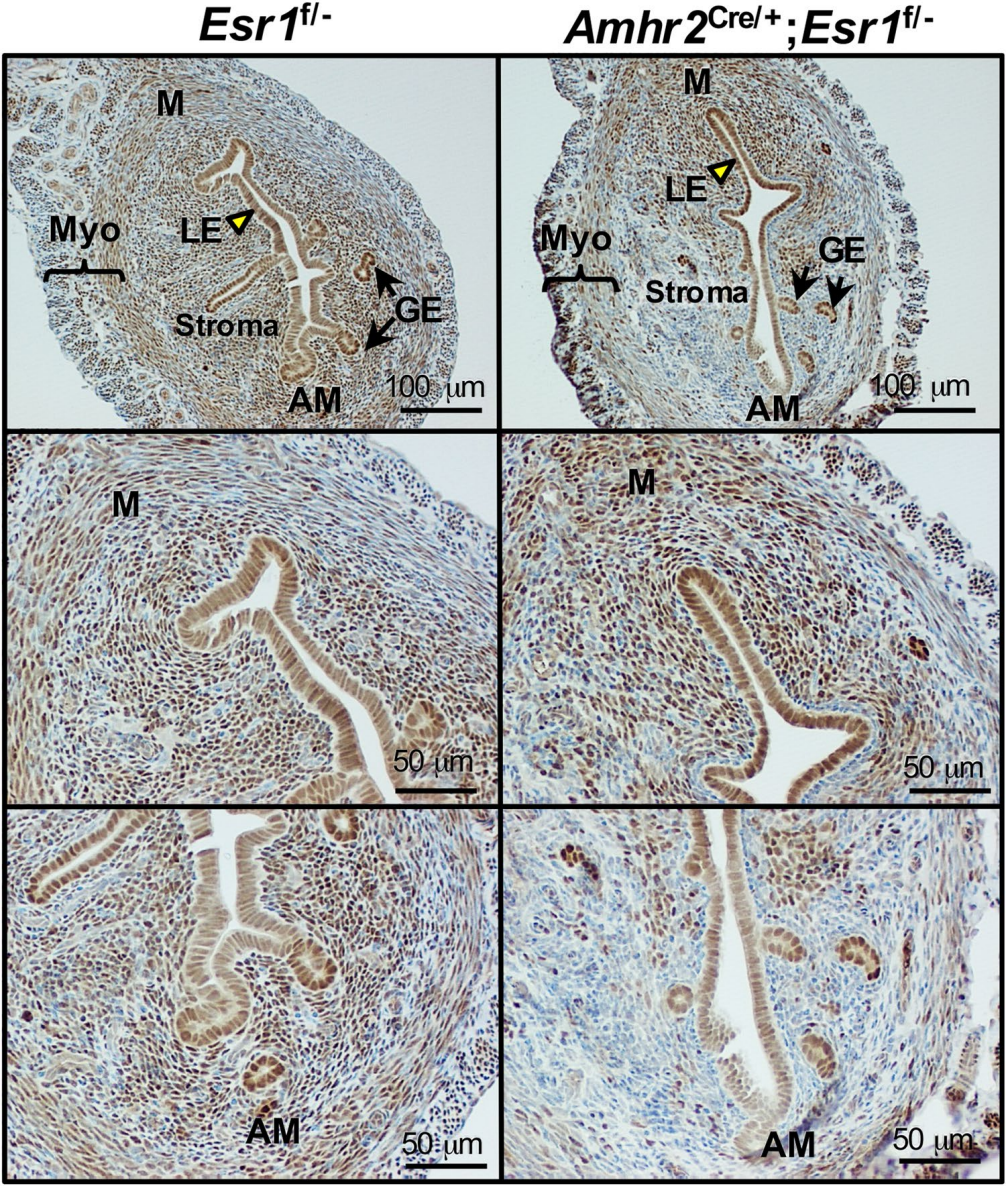
Selective deletion of ESR1 anti-mesometrial mouse uterine stromal cells. ESR1 deletion was confirmed using immunohistochemical (IHC) staining of ESR1 in whole uterine sections; mesometrium (M) and anti-mesometrium (AM), in ovarian intact adult (12-week-old) *Esr1* ^f/−^ and *Amhr2* ^Cre/+^; *Esr1* ^f/−^ females. LE = luminal epithelial cells, GE = glandular epithelial cells, and Myo = myometrium. Representative images shown.

*Amhr2* ^Cre/+^ mice were bred with *Esr1* ^f/−^ to specifically delete ESR1 in uterine stromal cells. Deletion of *Esr1* in the uterine tissues was confirmed using ESR1 immunohistochemical (IHC) analysis in 12-week-old mice. In the control uteri (*Esr1* ^f/−^), ESR1 protein was detected throughout uterine cross-sections, including epithelial, stromal, and muscle cell layers (Fig. 1). In the *Amhr2* ^Cre/+^; *Esr1* ^f/−^ uteri, ESR1 protein was ablated in the stromal cells of the uterine anti-mesometrial pole whereas the expression of ESR1 remained intact in the epithelial cell layer as well as in the stromal cells in the mesometrial pole (Fig. 1). We observed variable degrees of ESR1 deletion in individual *Amhr2* ^Cre/+^; *Esr1* ^f/−^ mice. This is likely due to uneven expression of the Cre-recombinase amongst stromal cells in the *Amhr2* ^Cre/+^ mouse line^18–20^. Therefore, the extent of deletion of ESR1 in the circular smooth muscle cells varied between individual animals (Supplementary Fig. S1).

**Figure 2:**
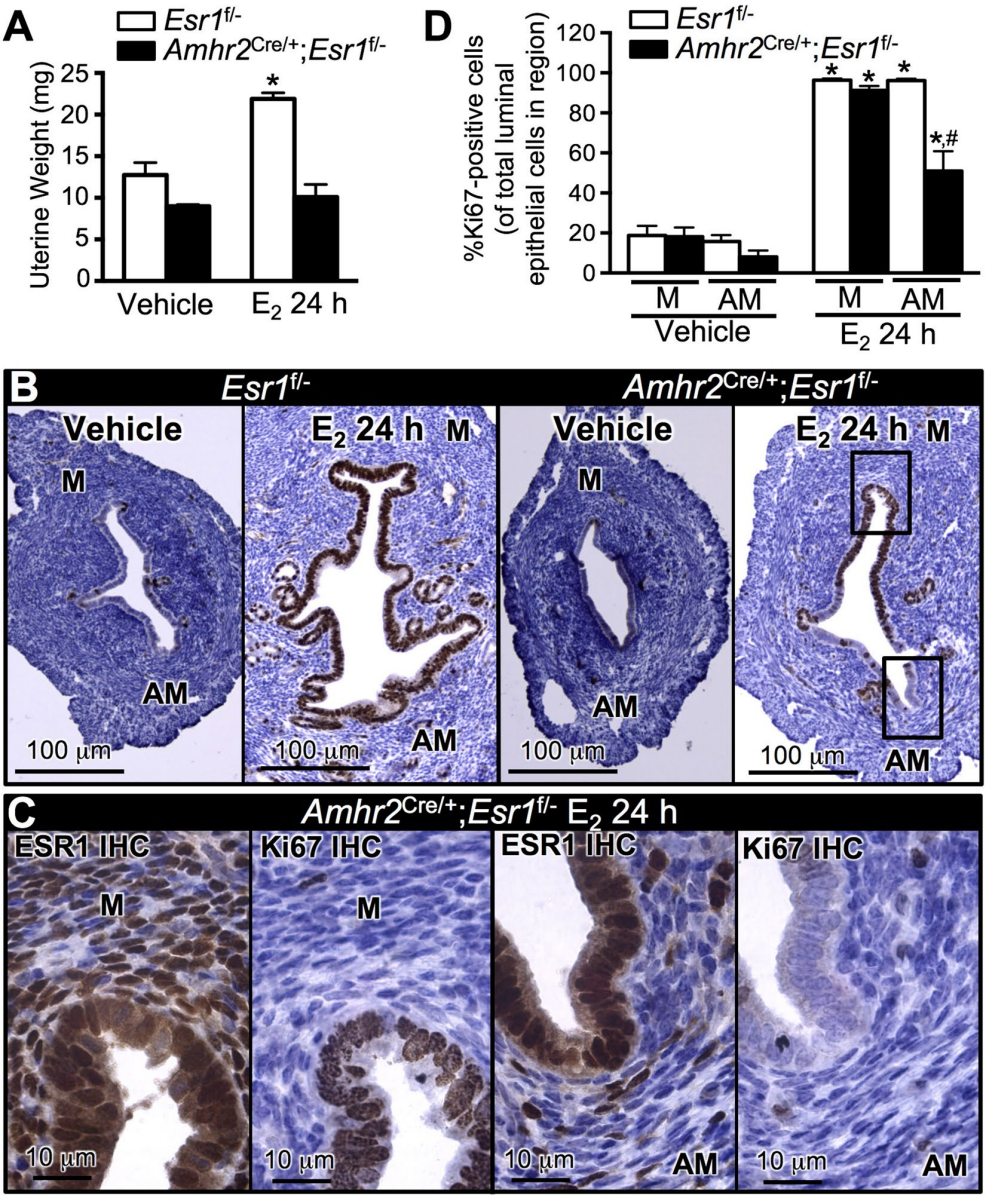
Uterine response to E_2_ treatment (24 h) in the absence of anti-mesometrial stromal ESR1. Adult (8–12-week-old) *Esr1* ^f/−^ and *Amhr2* ^Cre/+^; *Esr1* ^f/−^ females were ovariectomized and treated with vehicle or E_2_ for 24 h. (**A**) Uterine wet weight after 24 h of E_2_ treatment. *p < 0.05; significant difference between vehicle and E_2_ treated samples within genotype. N = 3 mice/genotype/treatment. (**B**) Uterine epithelial cell proliferation determined by Ki67 IHC staining in *Esr1* ^f/−^ and *Amhr2* ^Cre/+^; *Esr1* ^f/−^ uteri. (**C**) Higher magnification of *Amhr2* ^Cre/+^; *Esr1* ^f/−^ treated with E_2_ for 24 h. Uterine sections were stained with Ki67 and ESR1 antibodies in adjacent sections. Note that epithelial cell proliferation, as indicated by the appearance of Ki67, is primarily observed in the M where ESR1 is expressed in the adjacent stromal cells. (**D**) Percentage of Ki67-positive cells of total luminal epithelial cells in M vs. AM regions. *p < 0.05; significant difference between vehicle and E_2_ treated samples within genotype and region. ^#^ *p* < 0.05; significant difference between *Esr1* ^f/−^ and *Amhr2* ^Cre/+^; *Esr1* ^f/−^ uteri after E_2_ treatment in the AM region, unpaired *t-*test. N = 4–8 mice/genotype/treatment. All graphs represent mean ± SEM. M = Mesometrium, AM = Anti-mesometrium. Representative images shown.

Using our previous mouse model in which *Esr1* is selectively deleted in uterine epithelial cells, we reported that uterine epithelial ESR1 was not required for E_2_ to induce uterine epithelial cell proliferation^8^. No E_2_ induced epithelial proliferation occurs in global *Esr1*-null uteri^21^, demonstrating that uterine ESR1 is needed to mediate epithelial proliferation, thus we hypothesized that stromal ESR1 was required for paracrine regulation of epithelial cell proliferation. We collected the tissues 24 h after E_2_ treatment of ovariectomized 8–12-week-old females to observe E_2_-induced uterine wet weight increase, which reflects uterine growth (late response). In *Esr1* ^f/−^ uteri, E_2_ significantly increased the uterine wet weight compared to the vehicle treated controls (Fig. 2A). However, there was no uterine weight increase in the E_2_ treated *Amhr2* ^Cre/+^; *Esr1* ^f/−^ uteri compared to the vehicle control. Evaluation of cellular proliferative responses to E_2_, as reflected by expression of Ki67 proliferative marker, revealed few Ki67 positive cells in vehicle treated uteri of both genotypes. As expected, the luminal epithelial cells of E_2_ treated *Esr1* ^f/−^ uteri were positive for Ki67 (Fig. 2B). However, the luminal epithelial cells exclusively in the mesometrial but not in the anti-mesometrial pole of the E_2_ treated *Amhr2* ^Cre/+^; *Esr1* ^f/−^ uteri were positive for the Ki67 staining (Fig. 2B and higher magnification in Fig. 2C). A limited number of glandular epithelial cells were positive for Ki67 staining in both *Esr1* ^f/−^ and *Amhr2* ^Cre/+^; *Esr1* ^f/−^ uteri (Fig. 2B to C). Therefore, the Ki67-positive cells in the glandular epithelia were excluded from quantification of Ki67-positive cells. We found that the percentage of luminal epithelial cells that were Ki67-positive in the anti-mesometrial pole after 24 h E_2_ treatment was significantly less in *Amhr2* ^Cre/+^; *Esr1* ^f/−^ than in *Esr1* ^f/−^ uteri, whereas the percentage of epithelial cells that were proliferating in the mesometrial pole was similar in both *Amhr2* ^Cre/+^; *Esr1* ^f/−^ and *Esr1* ^f/−^ uteri (Fig. 2D). To determine whether the expression level of ESR1 in mesometrial and anti-mesometrial uterine stromal cells of *Amhr2* ^Cre/+^; *Esr1* ^f/−^ corresponds with the E_2_-induced proliferative response of epithelial cells, we compared ESR1 and Ki67 in adjacent sections. We found that ESR1 was ablated in anti-mesometrial stromal cells next to non-proliferating epithelial cells in *Amhr2* ^Cre/+^; *Esr1* ^f/−^ uteri (Fig. 2C and Supplementary Fig. S2). This finding suggests that loss of ESR1 in stromal cells prevents proliferation of immediately adjacent epithelial cells, indicating that signals emanating from stromal cells directly adjacent to responding epithelial cells transduce the required stimulus for proliferation. This stimulus is apparently unable to diffuse throughout the tissue, but rather works in a juxtacrine manner on neighboring epithelial cells.

### Loss of stromal ESR1 leads to blunted E_2_-induced cell cycle-related transcripts and proteins

**Figure 3:**
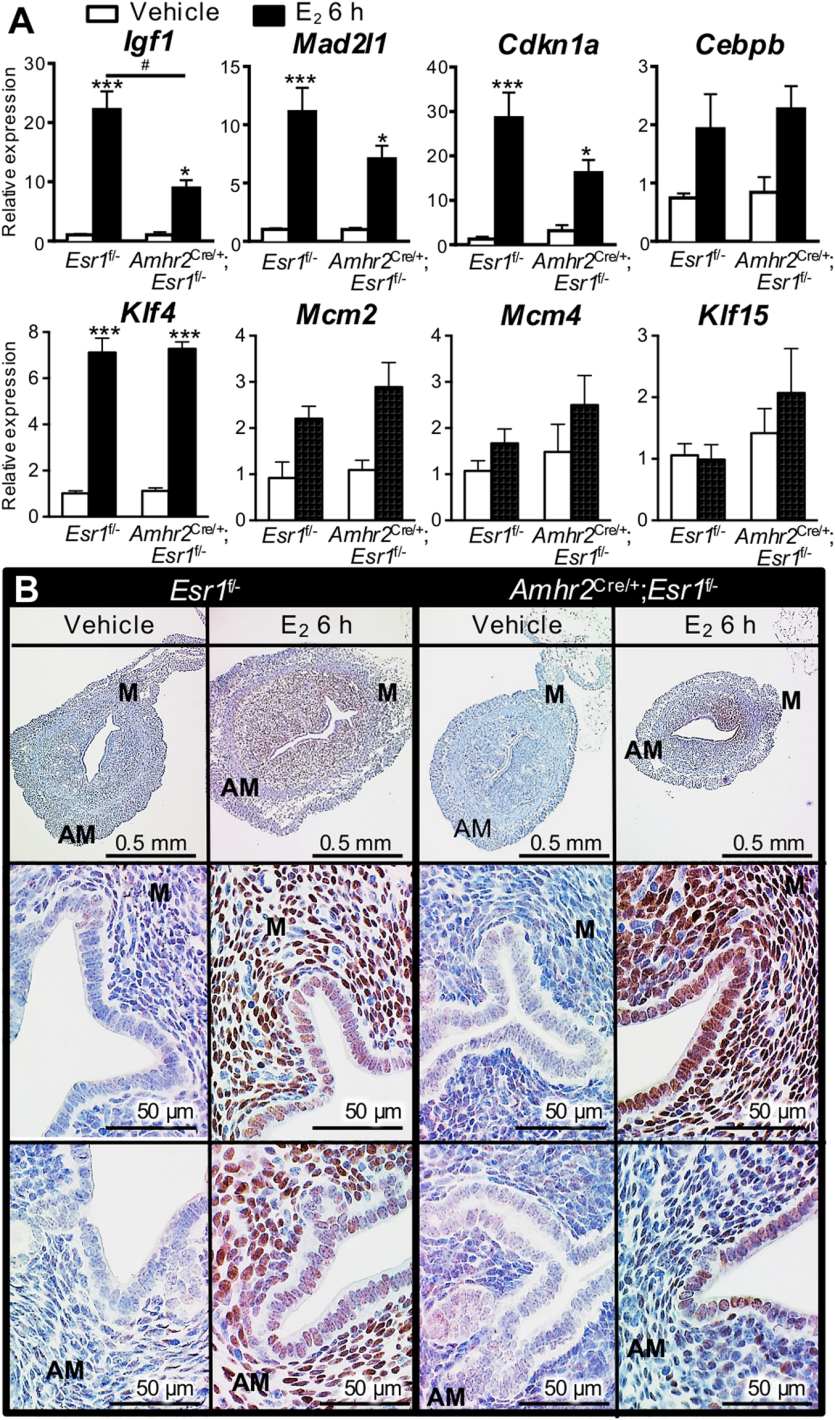
Cell proliferation-related uterine transcripts and protein in ovariectomized 8–12-week-old *Esr1* ^f/−^ and *Amhr2* ^Cre/+^; *Esr1* ^f/−^ females treated with E_2_ for 6 h. (**A**) Real-time PCR was performed and the relative expression values of *Igf1*, *Mad2l1*, *Cdkn1a*, *Cebpb*, *Klf4*, *Mcm2*, *Mcm4*, and *Klf15* were normalized to *Rpl7*. *, ***p < 0.05, 0.001; significant difference between vehicle and E_2_ treated samples within genotype. ^#^ *p* < 0.05; significant difference between E_2_ treated samples between genotype; unpaired *t-*test. (**B**) CEBPB protein expression after 6 h of E_2_ treatment in *Esr1* ^f/−^ and *Amhr2* ^Cre/+^; *Esr1* ^f/−^ uteri using IHC analysis. All graphs represent mean ± SEM. N = 3–5 mice/genotype/treatment. M = Mesometrium, AM = Anti-mesometrium. Representative images shown.

We previously reported that several genes associated with cell-cycle progression, including *Igf1*, CCAAT enhancer binding protein beta (*Cebpb*), cyclin-dependent kinase inhibitor 1a (*Cdkn1a*), and mitotic arrest deficient 2-like protein 1 (*Mad2l1*), were E_2_ responsive genes and that their expression was epithelial ESR1-independent, therefore, potentially mediated by stromal ESR1^8, 22^. To confirm that the expression of these transcripts and encoded proteins were stromal ESR1-dependent, we euthanized ovariectomized 8–12-week-old animals and collected the uterine tissues 6 h after the injection of E_2_. *Igf1*, *Mad2l1*, and *Cdkn1a* were significantly increased by E_2_ treatment compared to vehicle treated uteri in both *Esr1* ^f/−^ and *Amhr2* ^Cre/+^; *Esr1* ^f/−^ groups (Fig. 3A). However, the E_2_ induction of these transcripts in *Amhr2* ^Cre/+^; *Esr1* ^f/−^ uteri was blunted in comparison to the response of E_2_ treated *Esr1* ^f/−^ uteri; the *Igf1* was the most blunted (Fig. 3A). Mantena *et al*. have demonstrated that *Cebpb* is rapidly induced in uterine stromal cells by E_2_ and contributes to uterine epithelial cell proliferation^23^. Therefore, we reasoned that the deletion of stromal ESR1 would alter *Cebpb* expression in the uterus. However, we found that E_2_ induced similar levels of *Cebpb* transcript in *Esr1* ^f/−^ and *Amhr2* ^Cre/+^; *Esr1* ^f/−^ (Fig. 3A). To evaluate whether expression of the *Cebpb* gene in the whole uterus masked any differences in induction in stromal cells, expression of CEBPB protein was examined in uterine sections using IHC analysis. After E_2_ treatment, CEBPB was highly expressed in both epithelial and stromal cells in both the mesometrium and anti-mesometrium of the *Esr1* ^f/−^ uteri (Fig. 3B). In *Amhr2* ^Cre/+^; *Esr1* ^f/−^ uteri, CEBPB was highly induced in the mesometrial area, whereas the expression was minimally detected in the anti-mesometrial area (Fig. 3B).

In addition to these E_2_-responsive genes, other factors including kruppel like factor 4 (*Klf4*) and minichromosome maintenance complex components (*Mcm2* and *Mcm4*) are also involved in E_2_-induced uterine proliferation^24^. We found that *Klf4* was significantly induced by E_2_ treatment in both *Esr1* ^f/−^ and *Amhr2* ^Cre/+^; *Esr1* ^f/−^ uteri (Fig. 3A). However, *Mcm2* and *Mcm4* transcripts tended to be induced by E_2_ but not at significant levels. As expected, *Klf15* was not increased by E_2_ treatment as *Klf15* expression was previously shown to be regulated by P_4_^24^. These results suggest that uterine stromal ESR1 mediates the expression of some cell-cycle regulated genes and protein in response to E_2_ treatment.

**Figure 4:**
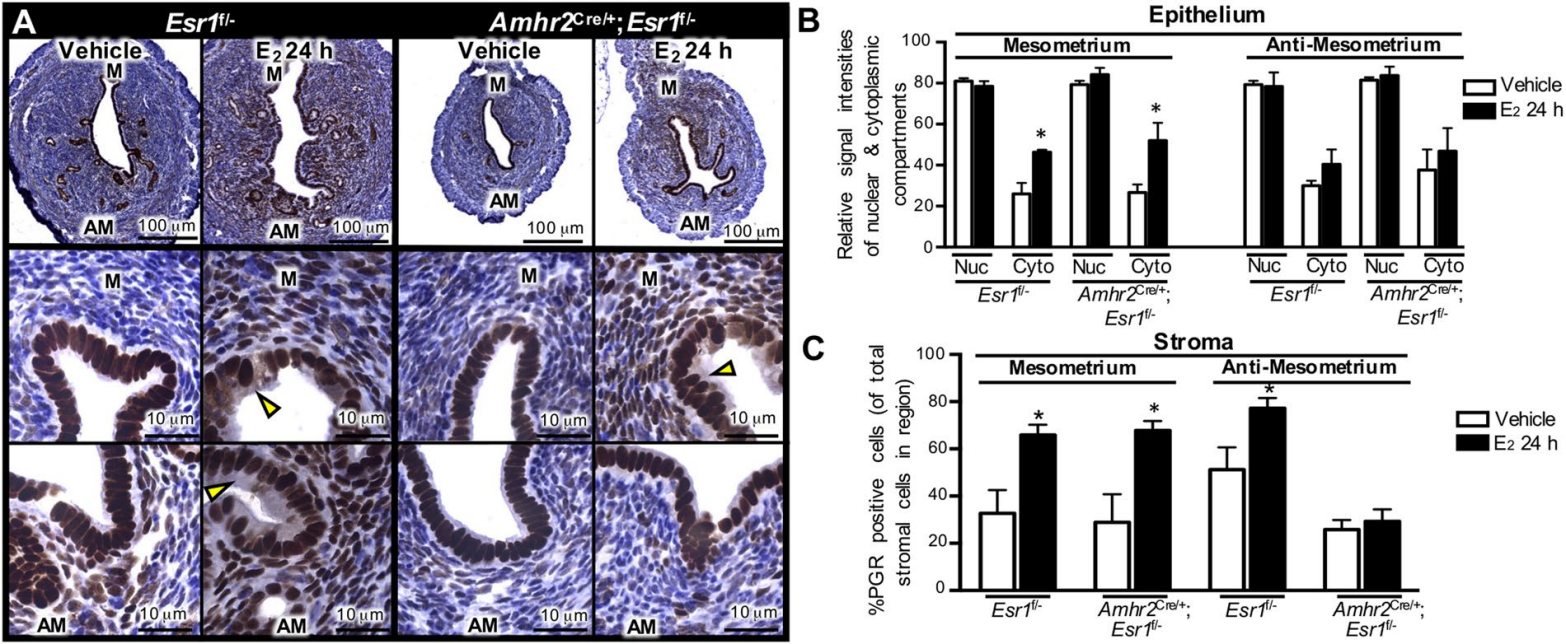
E_2_-induced progesterone receptor (PGR) expression in the uterus. Adult (8–12-week-old) female mice were ovariectomized and treated with vehicle or E_2_ for 24 h. (**A**) Top panel: Cross-sections of the whole uteri were stained with PGR antibodies. Bottom panels: PGR expression pattern in the mesometrial (M) vs. anti-mesometrial (AM) poles in *Esr1* ^f/−^ and *Amhr2* ^Cre/+^; *Esr1* ^f/−^ uteri. Representative images shown. (**B**) Relative signal intensities of nuclear (Nuc) and cytosolic (Cyto) compartments in the uterine luminal epithelial cells of *Esr1* ^f/−^ and *Amhr2* ^Cre/+^; *Esr1* ^f/−^ uteri after vehicle and E_2_ treatment for 24 h. (**C**) Percentage of PGR-positive cells of total stromal cells in M vs. AM regions. *p < 0.05; significant difference between vehicle and E_2_ treated samples within genotype and region. All graphs represent mean ± SEM. N = 3 mice/genotype/treatment.

We previously reported that deletion of ESR1 from epithelial cells had no effect on the expression of progesterone receptor (PGR), a hallmark E_2_-induced protein in the uterus (after 24 h of treatment^8^). We collected uterine tissues and evaluated the PGR protein levels using IHC analysis to determine how loss of anti-mesometrial stromal ESR1 affected uterine PGR expression. We found that E_2_ treatment compared to vehicle significantly increased PGR signal intensity in the cytoplasmic compartment in the mesometrial pole of both *Esr1* ^f/−^ and *Amhr2* ^Cre/+^; *Esr1* ^f/−^ animals (Fig. 4A; yellow arrowheads and Fig. 4B). In *Esr1* ^f/−^ uteri, E_2_ had a tendency to increase anti-mesometrial cytosolic PGR signal intensity (*p* = 0.2437). However, in the absence of anti-mesometrial stromal ESR1 in *Amhr2* ^Cre/+^; *Esr1* ^f/−^ animals, anti-mesometrial cytosolic PGR signal intensities in vehicle and E_2_ treatment were similar (*p* = 0.5765). In addition, the proportion of PGR-positive stromal cells was significantly increased in E_2_-treated *Esr1* ^f/−^ uteri in both mesometrial and anti-mesometrial poles (Fig. 4C). However, E_2_ treatment only increased PGR-positive cells in the mesometrial stromal cells of *Amhr2* ^Cre/+^; *Esr1* ^f/−^. From these findings, we conclude that ESR1 must be present in all uterine stromal cells for E_2_ to fully induce epithelial cell proliferation and properly regulate the pattern of PGR expression.

### Loss of stromal ESR1 in the uterus causes severe fertility defect

**Figure 5:**
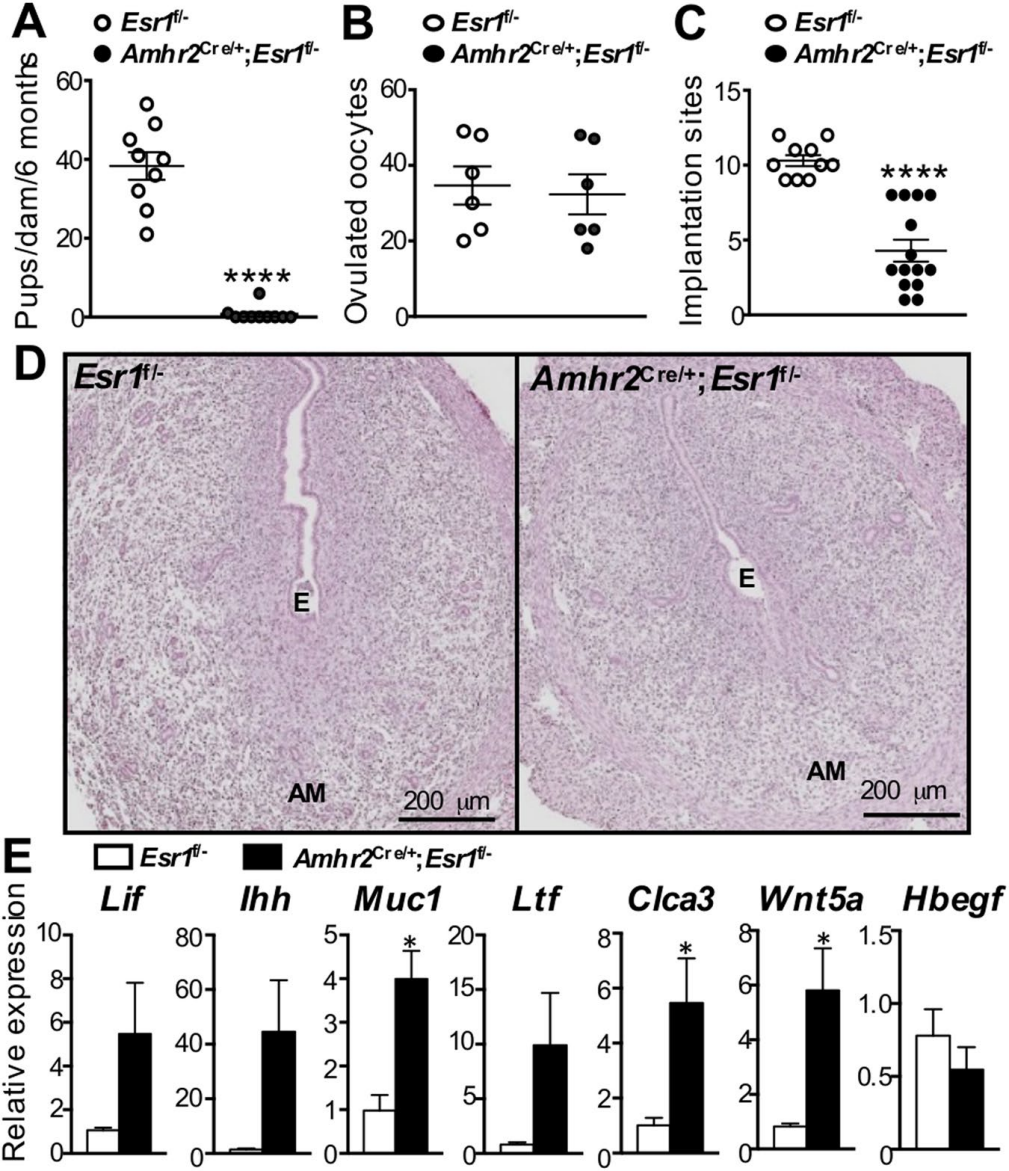
Fertility study of *Amhr2* ^Cre/+^; *Esr1* ^f/−^ and *Esr1* ^f/−^ mice. (**A**) Number of total pups per dam during 6-month breeding trial with wild-type males. Each data point represents an individual dam. ****p < 0.0001; unpaired *t-*test. N = 9–10 mice/genotype. (**B**) Ovulatory responses of 3–5-week-old females to gonadotropins indicated by total number of ovulated oocytes/mice. N = 6 mice/genotype. (**C**) Total number of 4.5 dpc implantation sites in 8–12-week-old female mice after natural mating. ****p < 0.0001; unpaired *t-*test. N = 3–4 mice/genotype. (**D**) Hematoxylin and eosin staining of implantation sites (4.5 dpc) in uterine cross sections. N = 3–4 mice/genotype. E = embryo, M = Mesometrium, AM = Anti-mesometrium. (**E**) Expression of implantation markers (*Lif*, *Ihh*, *Wnt5a*, and *Hbegf*) and E_2_-target transcripts (*Muc1*, *Ltf*, and *Clca3*) at 3.5 dpc in *Esr1* ^f/−^ and *Amhr2* ^Cre/+^; *Esr1* ^f/−^ 8–12-week-old female mice. *p < 0.05; unpaired *t-*test. N = 5–6 mice/genotype. All graphs represent mean ± SEM. Representative images shown.

To evaluate whether the anti-mesometrial stromal ESR1 is functionally required for female fertility, we determined the number of pups born to adult female mice during a 6-month period. The total number of pups delivered by *Amhr2* ^Cre/+^; *Esr1* ^f/−^ dams (0.7 ± 0.6 pups/dam) was significantly less than the number delivered by *Esr1* ^f/−^ dams (38.3 ± 3.5 pups/dam) (Fig. 5A). Eight out of ten *Amhr2* ^Cre/+^; *Esr1* ^f/−^ dams evaluated did not deliver any pups. Because *Amhr2* ^Cre/+^ was also expressed in the ovaries, we then investigated whether the subfertility phenotype in *Amhr2* ^Cre/+^; *Esr1* ^f/−^ females was due to impaired ovulation. Comparable numbers of oocytes were ovulated following gonadotropin stimulation of prepubertal *Esr1* ^f/−^ and *Amhr2* ^Cre/+^; *Esr1* ^f/−^ females (Fig. 5B). Since ovulation occurs normally, and our recent findings indicate that blastocyst development is not affected by the deletion of stromal ESR1 in the oviduct^25^, we reasoned that the subfertility phenotype in *Amhr2* ^Cre/+^; *Esr1* ^f/−^ females was due to impaired uterine function as a result of deletion of anti-mesometrial stromal ESR1. *Amhr2* ^Cre/+^; *Esr1* ^f/−^ adult females had significantly fewer implantation sites at 4.5 dpc, than *Esr1* ^f/−^ females (Fig. 5C). However, *Amhr2* ^Cre/+^; *Esr1* ^f/−^ implantation sites exhibited no apparent morphological defects (Fig. 5D).

To determine the potential cause of impaired implantation observed at 4.5 dpc, we evaluated several key uterine receptivity genes expressed during early gestation (3.5 dpc), at which time nidatory levels E_2_ are secreted to prepare the uteri for embryo implantation^26^. We found the hallmark implantation markers, *Lif* ^13^ and indian hedgehog (*Ihh*)^27^, tended to be expressed at higher levels in *Amhr*2^Cre/+^; *Esr1* ^f/−^ (*p* = 0.1237 and 0.0685, respectively) compared to *Esr1* ^f/−^ uteri (Fig. 5E). Surprisingly, significant elevation of E_2_-responsive genes such as mucin 1 (*Muc1*)^28^, lactotransferrin (*Ltf*)^28^, and chloride channel calcium-activated 3 (*Clca3*)^29^ was observed in *Amhr*2^Cre/+^; *Esr1* ^f/−^ compared to *Esr1* ^f/−^ uteri. These transcripts are normally suppressed during the 3.5 dpc pre-implantation period. Proper *Wnt5a* expression levels are crucial for embryo homing and optimal implantation^30^. Here, we demonstrated that *Wnt5a* transcript was significantly elevated in *Amhr*2^Cre/+^; *Esr1* ^f/−^ compared to *Esr1* ^f/−^ uteri at 3.5 dpc. However, heparin-binding EGF-like growth factor (*Hbegf*, another implantation marker^31^) was expressed at comparable levels in *Amhr*2^Cre/+^; *Esr1* ^f/−^ and *Esr1* ^f/−^ uteri. These findings indicate that stromal ESR1 in the anti-mesometrium is required for the suppression of some E_2_-regulated transcripts during implantation. However, when embryos did implant in *Amhr*2^Cre/+^; *Esr1* ^f/−^ uteri, implantation sites appear normal.

### Stromal ESR1 ablation contributes to a lack of uterine stromal cell proliferation

**Figure 6:**
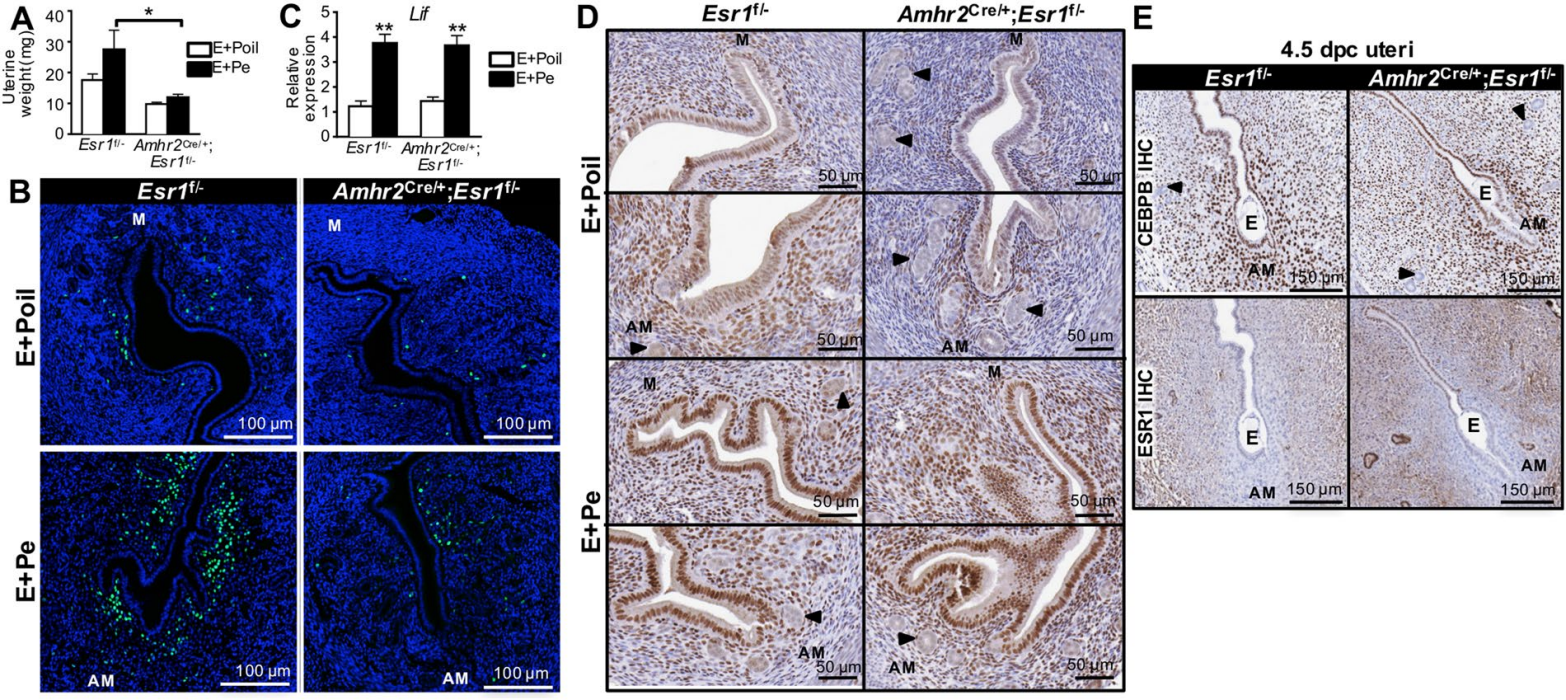
Proliferation of the uterine stromal cells after a series of E_2_ and P_4_ treatments (E+Pe) to mimic the hormonal profile during implantation and at 4.5 dpc. (**A**) Uterine wet weights of *Esr1* ^f/−^ and *Amhr2* ^Cre/+^; *Esr1* ^f/−^ 8–12-week-old females mice treated with E + Poil or E + Pe. *p < 0.05; significant difference between E+Pe treated *Esr1* ^f/−^ vs. *Amhr2* ^Cre/+^; *Esr1* ^f/−^ females. (**B**) EdU incorporation assay of *Esr1* ^f/−^ and *Amhr2* ^Cre/+^; *Esr1* ^f/−^ female mice that were treated with E+Poil or E+Pe. Cells with green signal represent EdU positive (DNA synthesis) cells. Cells with blue signal represent the nuclei stained with Hoescht. (**C**) Real-time PCR analysis of *Lif*. Values were normalized to *Rpl7*. **p < 0.01; significant difference between E+Poil and E+Pe treated samples within genotype. All graphs represent mean ± SEM. (**D**) CEBPB IHC staining of *Esr1* ^f/−^ and *Amhr2* ^Cre/+^; *Esr1* ^f/−^ female mice treated with E+Poil or E+Pe. Arrowheads indicate glandular epithelial cells. N = 4–6 mice/genotype/treatment. M = Mesometrium, AM = Anti-mesometrium. (**E**) Expression of CEBPB and ESR1 proteins of implantation sites from *Esr1* ^f/−^ and *Amhr2* ^Cre/+^; *Esr1* ^f/−^ uterine cross sections at 4.5 dpc using IHC analysis. Representative images shown. N = 3–4 mice/genotype. E = embryo.

Successful embryo implantation requires the cessation of uterine epithelial cell proliferation and subsequent stromal cell proliferation^32^. Because we observed impaired uterine receptivity, we investigated whether lacking ESR1 in the stromal cells contributed to defective uterine stromal cell proliferation or prevention of the cessation of the epithelial cell proliferation. To mimic the hormonal profile during embryo implantation, 8–12-week-old animals were ovariectomized and treated with E+Pe (see Methods for detail). Mice lacking anti-mesometrial stromal ESR1 did not show a uterine weight increase after E+Pe treatment (Fig. 6A). Moreover, uterine stromal cell proliferation was blunted in the absence of stromal ESR1 in the anti-mesometrial region (Fig. 6B). However, the uterine epithelial cells ceased proliferation similarly in *Esr1* ^f/−^ and *Amhr2* ^Cre/+^; *Esr1* ^f/−^ E+Pe treated uteri (Fig. 6B). In the absence of stromal ESR1, *Lif* expression was similarly induced in *Esr1* ^f/−^ and *Amhr2* ^Cre/+^; *Esr1* ^f/−^ E+Pe treated uteri (Fig. 6C). We then evaluated whether a lack of stromal cell proliferation was due to a loss of CEBPB expression. In the E+Poil control treatment group, there were fewer cells expressing CEBPB protein in the *Amhr2* ^Cre/+^; *Esr1* ^f/−^ compared to *Esr1* ^f/−^ uteri (Fig. 6D). However, upon E+Pe treatment, CEBPB was expressed similarly between *Esr1* ^f/−^ and *Amhr2* ^Cre/+^; *Esr1* ^f/−^ uteri (Fig. 6D). These results suggest that anti-mesometrial stromal ESR1 is required for the stromal cell proliferation and defective stromal cell proliferation is not due to a lack of CEBPB expression.

In addition, we evaluated 4.5 dpc implantation sites from 8–12-week-old mice to determine whether stromal ESR1 deletion in the anti-mesometrium altered CEBPB expression. At 4.5 dpc, CEBPB is expressed homogenously in the uterine epithelial and stromal cells surrounding the implantation sites (Fig. 6E), regardless of the expression of ESR1 in the stroma. Expression of ESR1 was not detected in the primary decidual zone in either *Esr1* ^f/−^ or *Amhr2* ^Cre/+^; *Esr1* ^f/−^ uteri. These results indicate that CEBPB is expressed independently from ESR1 expression in the implantation sites and that the implantation defect observed in the *Amhr2* ^Cre/+^; *Esr1* ^f/−^ females is not due to a lack of CEBPB.

### Ablation of anti-mesometrial stromal ESR1 caused impaired uterine decidual response after artificial stimulation

**Figure 7:**
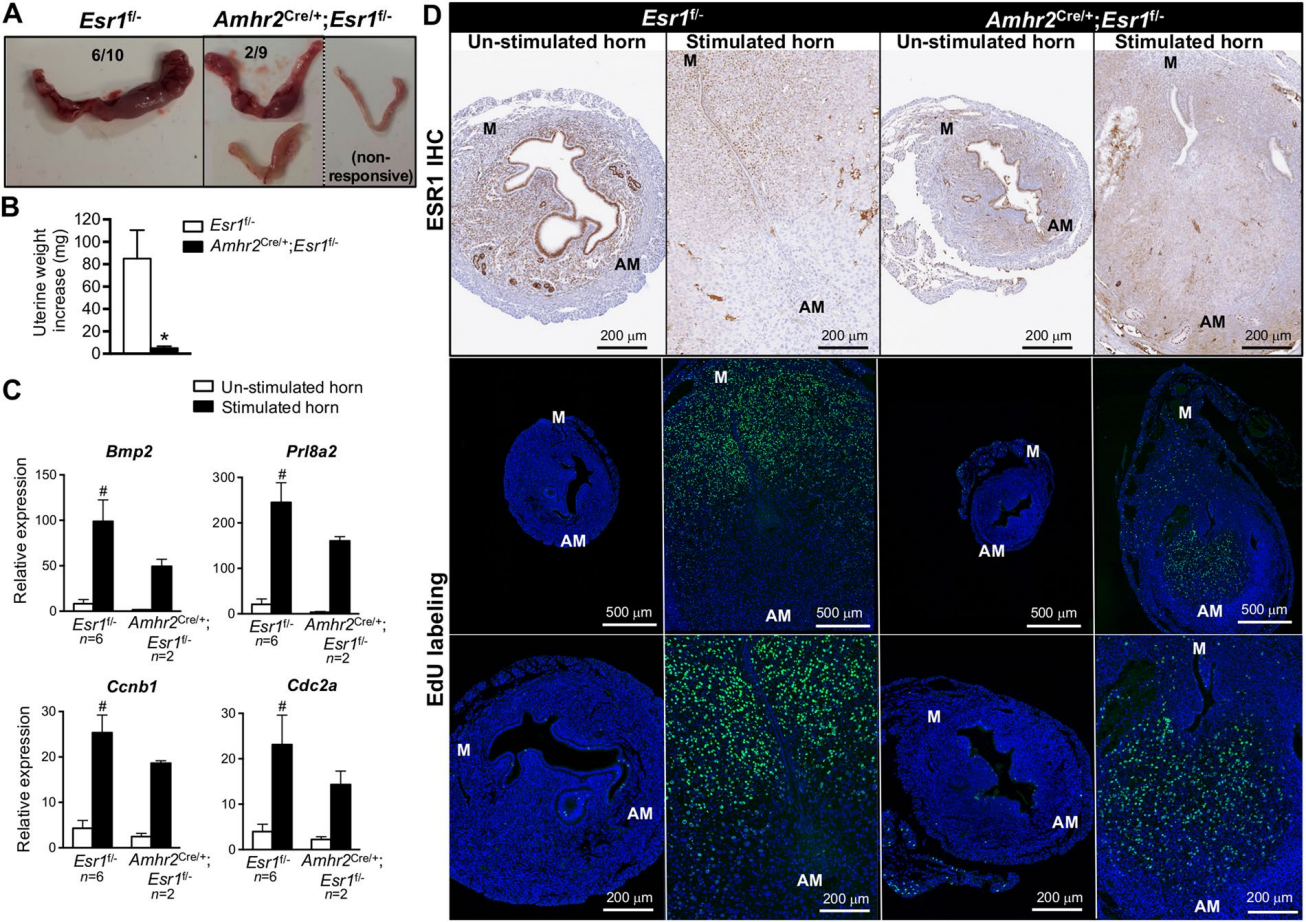
Impaired decidual response to artificial stimulation in the absence of stromal ESR1 in the anti-mesometrium. Artificial stimulation was used to induce decidual response in adult (8–12-week-old) *Esr1* ^f/−^ and *Amhr2* ^Cre/+^; *Esr1* ^f/−^ female mice (details for the treatment regime are described in Methods). (**A**) Gross morphology of uteri showing un-stimulated vs. stimulated uterine horns. Data below indicate the numbers of animals responding to the stimulation in *Esr1* ^f/−^ (6/10) or *Amhr2* ^Cre/+^; *Esr1* ^f/−^ (2/9) animals. Non-responsive *Amhr2* ^Cre/+^; *Esr1* ^f/−^ uteri (7/9) are also shown. (**B**) Uterine weight increased in the stimulated horns compared to un-stimulated horns (N = 9–10 animals/group). *p < 0.05, significant difference, unpaired *t*-test. (**C**) Transcript levels of decidual markers and cell-cycle regulators (*Bmp2*, *Prl8a2*, *Ccnb1*, and *Cdc2a*) in *Esr1* ^f/−^ (N = 6 of 10 animals) vs. *Amhr2* ^Cre/+^; *Esr1* ^f/−^ (N = 2 of 9 animals) uteri that responded to artificial stimulation. ^*#*^ *p* < 0.05, significant difference between un-stimulated and stimulated horns within genotype, unpaired *t*-test. All graphs represent mean ± SEM. (**D**) Uterine cross-section of *Esr1* ^f/−^ and *Amhr2* ^Cre/+^; *Esr1* ^f/−^ mice after artificial stimulation. Images illustrate ESR1 IHC and EdU incorporation assay in un-stimulated and stimulated horns. Green indicates cells in S-phase of DNA synthesis. Blue represents Hoescht stained nuclei.

Pawar *et al*. have shown that a lack of epithelial ESR1 impairs decidualization^17^. To clarify whether anti-mesometrial stromal ESR1 was also required for uterine decidualization, we evaluated the decidual response using a well-established method of injection of inert oil into the uterine lumen to artificially stimulate decidualization^17^. In 8–12-week-old *Esr1* ^f/−^ animals, artificial stimulation resulted in decidualization of 6 out of 10 uteri, whereas 2 out of 9 *Amhr2* ^Cre/+^; *Esr1* ^f/−^ females responded (Fig. 7A). The uterine weight increase resulting from decidual stimulation was significantly greater in *Esr1* ^f/−^ than in *Amhr2* ^Cre/+^; *Esr1* ^f/−^ uteri (Fig. 7B). Decidual markers and cell-cycle regulated genes, including bone morphogenic protein 2 (*Bmp2*), prolactin family 8, subfamily a, member 2 (*Prl8a2*), cyclin B1 (*Ccnb1*), and cyclin dependent kinase A1 (*Cdc2a*), were significantly induced in stimulated horns compared to un-stimulated horns in *Esr1* ^f/−^ uteri (Fig. 7C). The expression of these genes tended to be increased in responding horns of *Amhr2* ^Cre/+^; *Esr1* ^f/−^ animals, however, statistical analysis could not be performed as only 2 of 9 *Amhr2* ^Cre/+^; *Esr1* ^f/−^ animals responded to the artificial stimulation. As we observed an impaired decidual response in *Amhr2* ^Cre/+^; *Esr1* ^f/−^ animals, we next evaluated whether stromal cell proliferation was affected by a lack of stromal ESR1 by using an EdU incorporation assay. EdU was mainly detected in the mesometrial pole of *Esr1* ^f/−^ uteri, whereas a smaller area of EdU incorporation was seen in the anti-mesometrial pole of the decidualized *Amhr2* ^Cre/+^; *Esr1* ^f/−^ uterus. (Fig. 7D). However, EdU incorporation was not or was minimally detected in stimulated non-responding *Amhr2* ^Cre/+^; *Esr1* ^f/−^ uteri (Supplementary Fig. S3).

**Figure 8:**
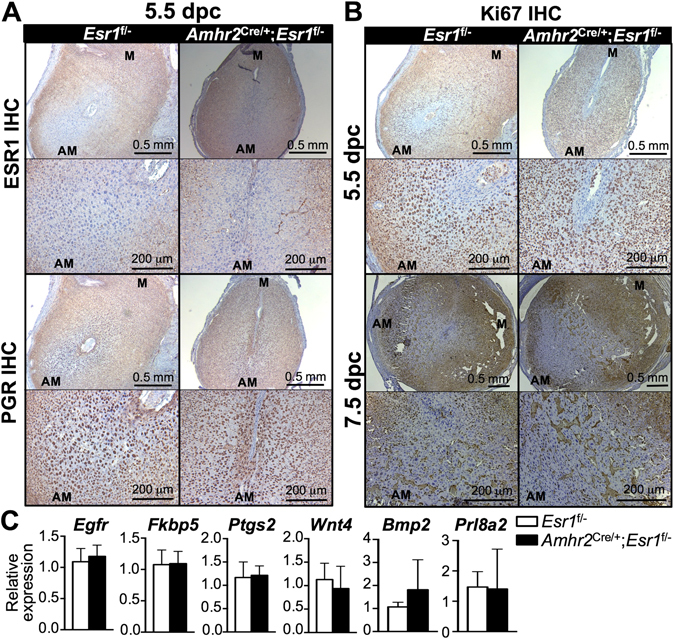
Expression of ESR1, PGR, Ki67 and marker genes for decidualization at 5.5 dpc and decidual cell proliferation at 7.5 dpc in the absence of anti-mesometrial stromal ESR1 in 8–12-week-old females. IHC analysis of (**A**) ESR1 and PGR and (**B**) Ki67 in *Esr1* ^f/−^ and *Amhr2* ^Cre/+^; *Esr1* ^f/−^ uteri collected at 5.5 and 7.5 dpc. N = 3–4 mice/genotype. (**C**) Transcript levels of decidualization markers in the uteri collected from decidual zones at 5.5 dpc, including *Egfr*, *Ptgs2*, *Pgr*, *Wnt4*, *Bmp2*, and *Prl8a2*. Graphs represent mean ± SEM. N = 3–4 mice/genotype. Representative images shown.

Because the uterus responds differently to artificial stimulation and embryo-initiated decidualization^33^, to determine whether stromal ESR1 is required for natural decidualization, we assessed the decidual response 5.5 and 7.5 dpc after natural mating of *Esr1* ^f/−^ and *Amhr2* ^Cre/+^; *Esr1* ^f/−^ adult females. We found that PGR was expressed similarly in *Amhr2* ^Cre/+^; *Esr1* ^f/−^ and *Esr1* ^f/−^ uteri regardless of ESR1 expression status in the anti-mesometrium (Fig. 8A). Moreover, proliferation of the decidual cells in the anti-mesometrium was comparable between *Esr1* ^f/−^ and *Amhr2* ^Cre/+^; *Esr1* ^f/−^ uteri at 5.5 and 7.5 dpc using Ki67 IHC analysis (Fig. 8B). There was no significant difference in expression of decidual gene markers, including epidermal growth factor receptor (*Egfr*), FK506 binding protein 5 (*Fkbp5*), prostaglandin-endoperoxide synthase 2 (*Ptgs2*), wingless-type MMTV integration site family, member 4 (*Wnt4*), *Bmp2*, and *Prl8a2* between *Esr1* ^f/−^ and *Amhr2* ^Cre/+^; *Esr1* ^f/−^ uteri at 5.5 dpc (Fig. 8C). These findings suggest that ESR1expression in anti-mesometrial stromal cells is required for normal decidualization in response to artificial stimulation, but that decidualization can occur after implantation.

### Loss of stromal ESR1 in uterine anti-mesometrium leads to an increased resorption of embryos post-decidualization

**Figure 9:**
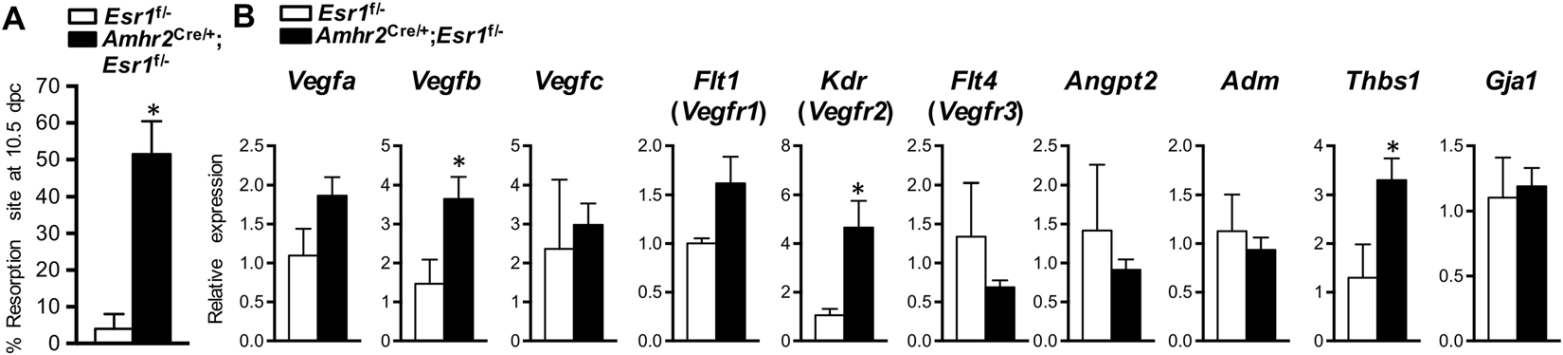
Resorption sites and the transcripts of angiogenesis markers at 10.5 dpc in *Esr1* ^f/−^ (N = 3) and *Amhr2* ^Cre/+^; *Esr1* ^f/−^ (N = 8) adult (8–12-week-old) female mice. (**A**) Percentage of resorption sites at 10.5 dpc in *Esr1* ^f/−^ vs. *Amhr2* ^Cre/+^; *Esr1* ^f/^uteri. (**B**) Transcript levels of angiogenic factors (*Vegfa*, *Vegfb*, *Vegfc*, *Flt1*, *Kdr*, *Flt4*, *Angpt2*, and *Adm*), anti-angiogenic factor (*Thbs1*), and gap junction protein alpha 1 (*Gja1*) of implantation sites at 10.5 dpc. *p < 0.05; unpaired *t*-test. All graphs represent mean ± SEM.

In the absence of stromal ESR1 in the anti-mesometrium, there was a 50% increase in resorption sites compared to *Esr1* ^f/−^ controls (Fig. 9A). To determine the cause of embryo resorption during this post-decidualization period of pregnancy, we assessed the expression of angiogenic genes in 10.5 dpc implantation sites. Several studies show that uterine decidual response is marked by an increase in uterine vasculature that subsequently provides nutrients to developing embryos^14^. Decidual vascularization is mainly regulated by vascular endothelial growth factors (VEGFs) and angiopoietins^34^. The hallmark features of decidual vascularization consist of angiogenic genes^34^, including *Vegfa*, *Vegfb*, *Vegfc*, FMS-like tyrosine kinase 1 (*Flt1*, also known as Vegf receptor 1 or *Vegfr1*), kinase insert domain protein receptor (*Kdr*, also known as *Vegfr2*), *Flt4* (or *Vegfr3*), angiopoietin 2 (*Angpt2*), adrenomedullin (*Adm*), anti-angiogenic factors such as thrombospondin (*Thbs1*)^35^, and gap junction protein alpha 1 (*Gja1*, also known as connexin 43)^36^. At 10.5 dpc, the implantation sites of *Amhr*2^Cre/+^; *Esr1* ^f/−^ showed significantly higher levels of *Vegfb*, *Kdr*, and *Thbs1* compared to those of *Esr1* ^f/−^ females, while other angiogenic genes (*Vegfa*, *Vegfc*, *Flt1*, *Flt4*, *Angpt2*, *Adm* and *Gja1*) were expressed at similar levels (Fig. 9B). This finding indicates that there is slight difference in the expression of angiogenic and anti-angiogenic markers in the absence of anti-mesometrial stromal ESR1, which may perturb optimal angiogenesis, leading to a failure of embryo development, and increased embryo resorption.

## Discussion

We report here that the expression of ESR1 in uterine stromal cells is necessary for E_2_-induced epithelial cell proliferation. Initially, based on the previous evidence that epithelial ESR1 regulated implantation and decidualization^8, 17, 22^, we hypothesized that stromal ESR1 was not necessary for embryo implantation and decidualization. However, the results presented in this study demonstrate that loss of stromal ESR1 caused over-expression of E_2_-regulated genes that are normally suppressed during early pregnancy to provide a receptive uterine environment, leading to decreased embryo implantation. Stromal ESR1 also appeared to be required for uterine decidual response to artificial stimulation. Additionally, we observed aberrant expression of some angiogenic factors at 10.5 dpc, which could lead to disrupted angiogenesis in the absence of stromal ESR1 in the anti-mesometrium. Together, our findings indicate that uterine epithelial cell proliferation is modulated by stromal ESR1 and that E_2_ orchestrates its function through both epithelial and stromal ESR1 in order to provide the optimal uterine environment for embryo implantation and uterine decidualization.

Consistent with previous findings, the Cre activity of Amhr2^Cre/+^ animals was only active in the anti-mesometrial pole of the uterus^18–20^, therefore, in *Amhr2* ^Cre/+^ *Esr1* ^f/−^ animals, the deletion of stromal ESR1 was observed only in the anti-mesometrium leaving the expression of stromal ESR1 in the mesometrium intact. Such a model system has exceptional specificity, since in the same animal only a portion of the tissue is affected by Cre expression, while other portions essentially function as an internal control. Note that we observed a higher level of ESR1 expression in anti-mesometrial epithelial cells than in mesometrial epithelial cells, however, only in some animals (3/8 *Amhr2* ^Cre/+^; *Esr1* ^f/−^ animals evaluated, Supplementary Fig. S1). This phenomenon might occur because luminal epithelial cells that developed in a region lacking stromal ESR1 were developmentally altered and had characteristics of glandular epithelial cells, which normally expressed more ESR1 than epithelial cells.

Along with several groups, our laboratory, has demonstrated that E_2_ mediates its proliferative effect through stromal ESR1 via paracrine activity by inducing secretion of growth factors, such as *Igf1*, and cell-cycle related proteins, including *Mad2l1*, *Cdkn1a*, and *Cebpb* ^7–9, 11, 22, 23, 37, 38^. These findings are consistent with our previous report that the ablation of epithelial ESR1 does not affect the expression level of these growth factors and cell cycle regulated genes^8^. However, in our stromal ESR1 deletion model, E_2_-induced *Igf1*, *Mad2l1*, *Cdkn1a*, and *Cebpb* were attenuated, but not absent. This discrepancy likely reflects selective deletion of anti-mesometrial stromal ESR1 and retention of mesometrial ESR1 to mediate the observed responses.

Here, we observed prominent epithelial cell proliferation in the mesometrium after E_2_ treatment, whereas the proliferation was blunted in the anti-mesometrium. These findings illustrate the local requirement of stromal ESR1 activity for epithelial proliferation, indicating a juxtacrine mechanism in which the stromal factors have a localized action and primarily affect neighboring epithelial cell. This unique responsive pattern in the *Amhr2* ^Cre/+^; *Esr1* ^f/−^ uteri was observed with Ki67, CEBPB, and PGR protein expression after E_2_ treatment. By selectively deleting ESR1 in the anti-mesometrium, we have created a unique tool, in which both positive and negative controls are present in the same tissue, to test the effect of stromal ESR1 in E_2_-regulated cell proliferation.

Our studies provide compelling data regarding the role of ESR1 during normal uterine proliferation. Such findings have the potential to advance understanding of abnormalities of the endometrium, such as endometriosis and endometrial cancer. It is well-established that endometrial cancer type I is estrogen-dependent, ESR1-positive, and is the most common form of endometrial cancer (>80% of the endometrial cancer cases). Moreover, IGF1 has been shown to be the major driver of endometrial hyperplasia progression and endometrial cancer formation in women^39^. Additionally, Ghazal *et al*. has recently demonstrated that estrogen increases IGF1R expression and subsequently induces stromal cell proliferation in endometrial tissues from women with endometriosis^40^. Our studies showed that local production of IGF1 was regulated by the stromal cells underlying luminal epithelial cells via the stimulation of estrogen signaling through stromal ESR1. Together, these findings advance general understanding regarding the roles of estrogen during normal endometrial growth. Furthermore, our studies indicate the potential source and location of growth factor production that could be targeted by therapeutic agents against endometrial growth abnormalities such as endometriosis, endometrial hyperplasia, and cancer.

Ablation of anti-mesometrial stromal ESR1 led to a decreased fecundity, without affecting the number of ovulated oocytes and blastocysts in the uterus^25^. Pawar *et al*. and our laboratory have demonstrated that epithelial ESR1 is necessary for embryo implantation^8, 17^. Implantation of embryos normally occurs exclusively in the anti-mesometrial pole of the uterus^12^. We found that ablation of anti-mesometrial stromal ESR1 affected uterine receptivity, partly due to increased expression of E_2_-regulated genes, such as *Muc1*, *Ltf*, and *Clca3*. This finding suggests that the stromal ESR1 is involved in regulating the gene expression in epithelial cells. Lacking stromal ESR1 in the anti-mesometrium decreases the number of implantation sites at 4.5 dpc by 50%. Cessation of uterine epithelial cell proliferation and increase stromal cell proliferation are also crucial steps for normal uterine receptivity^32^. Using a hormonal profile mimicking implantation (E+Pe), we found that lacking ESR1 in the anti-mesometrial stromal cells caused diminished stromal cell proliferation. Together, these findings suggest that *Amhr2* ^Cre/+^; *Esr1* ^f/−^ females are less receptive to embryo attachment/implantation due to a lack of stromal cell proliferation in the specific area of the uterus linked to the loss of anti-mesometrial stromal ESR1.

LIF, a key mediator of uterine receptivity, is expressed in glandular epithelial cells and regulated by epithelial ESR1^8, 13, 17^. Loss of glandular LIF expression impairs receptivity^13^. As expected, *Lif* transcript was comparable in both *Esr1* ^f/−^ and *Amhr2* ^Cre/+^; *Esr1* ^f/−^ uteri in the E+Pe model, but tended to be increased in the uterus at 3.5 dpc. This result suggests that ablation of stromal ESR1 does not significantly affect the production of *Lif* in the glandular epithelial cells. This comparable production of *Lif* in *Amhr2* ^Cre/+^; *Esr1* ^f/−^ and *Esr1* ^f/−^ uteri may facilitate the initiation of embryo attachment/implantation in the absence of proper stromal cell proliferation. However, uterine receptivity is not determined by the proliferation status of epithelial and stromal cells or the production of LIF alone, other factors such as HBEGF and WNT5A are also crucial implantation signals^30, 31^. We found that *Wnt5a* transcript was significantly increased in *Amhr2* ^Cre/+^; *Esr1* ^f/−^ uteri, which could potentially disrupt embryo homing and subsequently lead to implantation failure.

Mantena *et al*. have demonstrated that both E_2_ and P_4_ treatment regulate uterine CEBPB expression^23^. In their report, E_2_ rapidly increased uterine CEBPB expression in ovariectomized mice within 1 h of treatment whereas P_4_ induced expression after 24 h. However, the expression of CEBPB during decidualization on day 6 of pregnancy is solely regulated by PGR, as CEBPB protein is attenuated by a PGR antagonist (RU486). We also found rapid induction of CEBPB by E_2_, which is required for epithelial cell proliferation, but only in the mesometrial region, indicating that stromal ESR1 mediated the induction. However, CEBPB expression in the uterine anti-mesometrium as a result of E+Pe treatment or decidual response was independent of stromal cell ESR1 expression.

Previous findings using the original *Esr1* ^−/−^ mouse line^41^ showed that global deletion of ESR1 did not affect uterine decidual responses in an artificial decidualization model^42, 43^. However, our recent unpublished data using the Ex3αERKO mouse line^21^ indicates that global loss of *Esr1* prevents decidualization. Additionally, recent findings suggest that ESR1 in uterine epithelial cells is in fact modulating the decidualization process^17^. Protein expression analysis of ESR1 in mouse uteri during pregnancy clearly showed that ESR1 is not expressed in the primary decidual zone^44^, which suggests that stromal ESR1 in the uterine anti-mesometrium is not required for the decidual response. However, after artificial stimulation, uteri with stromal ESR1 deletion in the anti-mesometrium showed impaired decidual responses as measured by uterine weight increase and cell proliferation. These results confirm recent findings showing ESR1 is required for normal decidualization of cultured human stromal cells^45^.

From our findings, we surmise that the regulation of uterine epithelial cell proliferation in response to E_2_ mediated by ESR1 is through a local cell-cell communication between the stromal cells and adjacent epithelial cells. In addition, this communication is crucial for normal embryo attachment/implantation and decidual response to artificial stimulation.

## Methods

### Animals and experimental procedures

We generated a mouse model with stromal cell selective deletion of ESR1 (encoded by the *Esr1* gene) using *Amhr2* ^Cre/+^ animals^19, 46^ bred with our *Esr1* ^f/−^ animals^21^. Female *Esr1* ^f/−^ mice were considered control animals for experiments. *Amhr2* ^Cre/+^ animals exhibit higher expression levels of Cre activity in anti-mesometrial uterine stromal cells than in mesometrial uterine stromal cells^18–20^. Adult females (8–12-week-old) were ovariectomized and housed for 14 days to eliminate the endogenous circulating ovarian steroid hormones. Animals were randomly grouped and subcutaneously injected with vehicle control (sterile normal saline) or 17β-estradiol (E_2_, Steraloids, Newport, RI) at a dose of 0.25 μg/mouse in saline. To evaluate transcript and protein expression initially regulated by E_2_ (early responses), we euthanized the mice 6 h after the injection of E_2_ and collected the uterine tissues. To determine the E_2_-induced protein expression and uterine wet weight increase reflecting uterine growth (late responses), we collected the tissues 24 h after E_2_ treatment. In some experiments, animals were injected with E_2_ and P_4_ (Sigma) called “E+Pe” to mimic the hormonal profile during implantation as previously described^47^. The control group of this experiment was injected with the series of hormones similar to the E+Pe treatment group except the last nidatory dose of E_2_ was replaced with sesame oil, called “E+Poil”. The animals were injected intraperitoneally with 100 μL of 5-ethynyl-2-deoxyuridine (EdU, Invitrogen, Carlsbad, CA) at a dose of 2 mg/mL in phosphate-buffered saline (PBS) 2 h prior to sacrifice. At the time of collection, uteri were weighed, and one uterine horn was snap frozen and stored at −80 °C for RNA extraction. The contralateral horn was collected in 10% buffered formalin solution for histological analysis. Animals were handled according to National Institute of Environmental Health Sciences (NIEHS) Animal Care and Use Committee guidelines and in compliance with NIEHS-approved animal protocol. All methods were performed in accordance with the relevant guidelines and regulations.

### Artificial decidualization

Adult (8–12-week-old) female mice were ovariectomized (*n* = 9–10 animals/genotype). Two weeks after ovariectomy, the mice were treated with E_2_ (100 ng/mouse) subcutaneously for 3 consecutive days (Day 1 or D1) to D3. On D6-D11, the mice were treated daily with P_4_ (1 mg/mouse) together with E_2_ (6.7 ng/mouse). Artificial decidualization was stimulated on D8 by a intraluminal injection of 50 μL of sesame oil into the right uterine horn, the left horn was not injected to be used as a negative control. EdU was injected intraperitoneally 2 h before sacrifice. The animals were euthanized on D11 (72 h after intraluminal oil injection). The uteri (decidualized and non-decidualized horns) were weighed and fixed in formalin for histological analysis. Sections of uteri were snap frozen for RNA analysis. Uterine weight change reflects the weight increase in decidualized horns compared to non-decidualized horns. Six of ten *Esr1* ^f/−^ females responded to decidual stimulation, whereas two of nine *Amhr2* ^Cre/+^; *Esr1* ^f/−^ females responded. Therefore, only uteri from 6 *Esr1* ^f/−^ and 2 *Amhr2* ^Cre/+^; *Esr1* ^f/−^ animals were included for the RNA data analysis.

### Fertility study and collection of implantation sites

To evaluate the ovulatory response, pubertal (3–5-week-old) *Esr1* ^f/−^ and *Amhr2* ^Cre/+^; *Esr1* ^f/−^ females were injected with 5 U of pregnant mare’s serum gonadotropin (PMSG, EMD Millipore, Billerica, MA) in sterile normal saline. Human chorionic gonadotropin (hCG, EMD Millipore) was injected 48 h after PMSG injection. At 18 h post hCG injection, the ovulated oocytes were collected from the oviduct, the number of oocytes were counted and recorded. In the fertility study, adult (8-week-old) *Esr1* ^f/−^ and *Amhr2* ^Cre/+^; *Esr1* ^f/−^ females were mated with a male proven breeder (C57BL6/J, Jackson Laboratory) continuously for 6 months. Numbers of pups per litter per dam over the 6-month period were recorded.

To collect the uteri at different stages of pregnancy as well as implantation sites, adult (8–12-week-old) *Esr1* ^f/−^ and *Amhr2* ^Cre/+^; *Esr1* ^f/−^ females were mated with the stud male (B6/D2F1/J, Jackson Laboratory) over night. The next morning, the observed presence of a copulatory plug was designated as 0.5 dpc. At 3.5, 4.5, 5.5, 7.5 and 10.5 dpc, uteri were collected from both *Esr1* ^f/−^ and *Amhr2* ^Cre/+^; *Esr1* ^f/−^ mice. To visualize implantation sites at 4.5 dpc, Chicago Blue dye (Sigma) was injected into the tail vein as previously described^48^. The visible blue bands indicate the implantation sites. Some of the implantation sites were collected for RNA extraction, the rest of the implantation sites were collected in formalin for histological analysis. At 5.5, 7.5 and 10.5 dpc, the implantation sites are visible without the blue dye injection. The sites were collected for RNA and histological analysis.

### Real-time RT-PCR analysis

After collection, RNA was extracted from uteri (or implantation sites) using TriZol reagent (Invitrogen) according to manufacturer’s protocol. Genomic DNA contamination was eliminated by incubating the RNA samples with DNaseI (Invitrogen). Two μg of RNA was used as a template for cDNA synthesis using SuperScript II (Invitrogen). Real-time PCR and analysis was performed as described previously^8^. Expression values were normalized to ribosomal protein L7 (*Rpl7*) and calculated as fold change over vehicle control (or over E+Poil) of the *Esr1* ^f/−^ group. The primer sequences of *Rpl7*, *Lif* (Leukemia inhibitory protein), *Ihh* (indian hedgehog), *Igf1* (insulin-like growth factor-1), *Mad2l1* (mitotic arrest deficient-like 1), *Cdkn1a* (Cyclin-Dependent Kinase Inhibitor 1 A), *Cebpb* (CCAAT Enhancer Binding Protein Beta), *Klf4* and *Klf15* (Kruppel like factors), *Mcm2* (minichromosome maintenance complex component 2), and *Ltf* (lactotransferrin) were reported previously^8, 22^. The primer sequences for *Adm* (adrenomedullin), *Antpt2* (angiopoietin 2), *Bmp2* (bone morphogenic protein 2), *Ccnb1* (cyclin B1), *Cdc2a* (cyclin dependent kinase A1), *Clca3* (chloride channel calcium-activated 3), *Egfr* (epidermal growth factor receptor), *Fkbp5* (FK506 binding protein 5), FMS-like tyrosine kinase 1 (*Flt1* also known as vascular endothelial growth factor receptor 1 or *Vegfr1*), *Flt4* (or *Vegfr3*), *Gja* (gap junction protein alpha), *Hbegf* (heparin-binding epidermal growth factor), *Kdr* (kinse insert domain protein receptor also known as *Vegfr2*), *Muc1* (mucin 1), *Prl8a2* (prolactin family 8, subfamily a, member 2), *Ptgs2* (prostaglandin-endoperoxide synthase 2), *Thbs1* (thrombospondin 1), vascular endothelial growth factors (*Vegfa*, *Vegfb*, and *Vegfc*), *Wnt4* and *Wnt5a* (wingless-type MMTV integration site family, member 4 and 5a, respectively) are as followed (5′ → 3′): *Adm*-F: CATCCAGCAGCTACCCTACG, *Adm*-R: TTCGCTCTGATTGCTGGCTT, *Antpt2-*F: TCGCTGGTGAAGAGTCCAAC, *Antpt2-*R: GTCAAACCACCAGCCTCCTG, *Bmp2-*F: GACTGCGGTCTCCTAAAGGTC G, *Bmp2-*R: CTGGGGAAGCAGCAACACTA, *Ccnb1-*F: TTGTGTGCCCAAGAAGATGCT, *Ccnb1-*R: GTACATCTCCTCATATTTGCTTGCA, *Cdc2a-*F: GGACGAGAACGGCTTGGAT, *Cdc2a-*R: GGCCATTTTGCCAGAGATTC, *Clca3-*F: AACAACGGCTATGAGGGCAT, *Clca3*-R: TGAGTCACCATGTCCTTTATGTGT, *Egfr*-F: GCATCATGGGAGAGAACAACA, *Egfr*-R: CTGCCATTGAACGTACCCAGA, *Fkbp5*-F: CCTCGCAGCCTTCCTGAAC, *Fkbp5*-R: CACTCCACGGCTTTGTTGTACTC, *Flt1*-F: GTGTCTATAGGTGCCGAGCC, *Flt1*-R: CGGAAGAAGACCGCTTCAGT, *Flt3*-F: CCGCAAGTGCATTCACAGAG, *Flt3*-R: TCGGACATAGTCGGGGTCTT, *Gja*-F: AGTGAAAGAGAGGTGCCCAGA, *Gja-*R: AATGAAGAGCACCGACAGCC, *Hbegf-*F: TTCTGGCCGCAGTGTTGTC, *Hbegf*-R: CTGAGCACGATCACCTCCC, *Kdr*-F: GCATACCGCCTCTGTGACTT, *Kdr*-R: AAATCGCCAGGCAAACCCAC, *Muc1-*F: CCCCTATGAGGAGGTTTCGG, *Muc1*-R: CAGATCAGAGTGCAGGGGTC, *Ptgs2*-F: AAGGCTCAAATATGATGTTTGCATT, *Prl8a2-*F: AAACACTTGTTTCACGCATGTATAG, *Prl8a2-*R: AGGAGTGATCCATGCACCCA, *Ptgs2*-R: CCCAGGTCCTCGCTTATGATC, *Wnt4*-F: AGTGACAAGGGCATGCAGC, *Thbs1*-F: CCTCCCCTCTGCTTTCACAA, *Thbs1*-R: TAACCGAGTTCTGGCAGTGAC, *Vegfa*-F: TATTCAGCGGACTCACCAGC, *Vegfa*-R: AACCAACCTCCTCAAACCGT, *Vegfb*-F: AGCTGACATCATCCATCCCAC, *Vegfb*-R: CAGCTTGTCACTTTCGCGG, *Vegfc*-F: GTGCTTCTTGTCTCTGGCGT, *Vegfc*-R: TTCAAAAGCCTTGACCTCGC, and *Wnt4*-R: CATCCTGACCACTGGAAGCC, *Wnt5a*-F: CGTGGTGTGAATGAACTGGG, *Wnt5a*-R: CCAAATGTGGGCGTGATTGT.

### Immunohistochemical (IHC) analysis

Formalin-fixed tissues were cross-sectioned (5 microns). The tissues were stained with mouse primary antibodies against ESR1 (ImmunoTech, Beckman Coulter, Pasadena, CA, #1545), PGR (ImmunoTech #1546), and Ki67 (BD Pharmingen, BD Biosciences, San Jose, CA, #550609) as indicated previously^22^. CEBPB (SC150, Santa Cruz Biotechnology, Dallas, TX) was stained at a concentration of 1:100 antibody diluted in blocking reagent containing 1% non-fat milk (Santa Cruz Biotechnology), 1% bovine serum albumin (Sigma), and 10% normal goat serum (Jackson ImmunoResearch, West Grove, PA) in automation buffer (BioCare Medical, Concord, CA). Detection of EdU incorporation for DNA synthesis in the uteri was performed as described previously^49^.

### Quantification analysis for Ki67- and PGR-positive cells

The images were taken using bright field microscope (DMi8, Leica Microsystems, Buffalo Grove, IL) with 10x and 100x objective lenses using Leica Application Suite (Leica Microsystems). Ki67- (10x) and PGR- (100x) positive cells were quantified using ImageJ software with Cell Counter Plugins. The Ki67-positive epithelial cells were counted and calculated as a percentage of total epithelial cells. The glandular epithelial cells were excluded as the majority of the glands are embedded within the stroma of the anti-mesometrial pole. As a result, the proliferation rate of glandular epithelial cells may vary based on the degree of ESR1 deletion in each animal. The total epithelial cell count ranged from 45–324 cells per microscopic field from a total of 3–8 animals per genotype. To eliminate bias from selecting the compartment that does or does not express ESR1, we instead calculated the Ki67-positive luminal epithelial cells of the entire mesometrial pole (top 50% of the uterus) and anti-mesometrial pole (bottom 50% of the uterus), regardless of ESR1 expression status (dotted lines indicated in Supplementary Fig. S2A).

The stromal cells with brown staining (PGR-positive) and blue staining (hematoxylin or PGR-negative) were counted per microscopic field by two different individuals. The number of brown and blue cells were summed as total stromal cells per microscopic field. The PGR-positive cells were calculated as a percentage of the total stromal cells. The total cells counted ranged from 131–285 cells per microscopic field from a total of 3 animals/genotype/treatment.

### Measurement of relative signal intensity of PGR in nuclear and cytosolic compartments

All images were taken with a 100x objective lens with similar and software settings. To quantify the light intensities of the nuclear and cytoplasmic compartment, the RGB images were converted into grayscale 8-bit images using ImageJ software and then were inverted. The darkest areas were converted to light white areas, and vice versa (color values from black to white = 0–255). The value of grayscale value was measured in the nuclear or cytoplasmic compartments of the epithelial cells using the wand tool to select an area of 1 × 1 pixel for each compartment within the microscopic field. The relative intensities were calculated as a percentage relative to 255-white signal (255 white signal = 100% relative intensity). The total cells counted ranged from 15–67 cells per image from a total of 3 animals/genotype/treatment.

### Statistical analysis

All graphs represent mean ± SEM. Statistical analysis was performed using GraphPad Prism version 6.0 h for Mac OS X (GraphPad Software, Inc., La Jolla, CA). Statistical significance is considered when *p* < 0.05 using two-way ANOVA with Tukey post hoc test, unless otherwise indicated.

## Electronic supplementary material

Supplementary information
