# Supplementary material for: Juxtacrine Activity of Estrogen Receptor α in Uterine Stromal Cells is Necessary for Estrogen-Induced Epithelial Cell Proliferation
Source: Sci Rep. 2017 Aug 21;7:8377. doi: 10.1038/s41598-017-07728-1 (PMC5566397; doi:10.1038/s41598-017-07728-1)
Supplement: Supplementary file 1 — Supplementary information [file 41598_2017_7728_MOESM1_ESM.pdf]

Supplementary Materials

Title: Juxtacrine Activity of Estrogen Receptor α in Uterine Stromal Cells is Necessary for Estrogen-Induced Epithelial Cell Proliferation

Authors: Wipawee Winuthayanon, Sydney L. Lierz, Karena C. Delarosa, Skylar R. Sampels, Lauren J. Donoghue, Sylvia C. Hewitt, and Kenneth S. Korach

Supplemental Figures

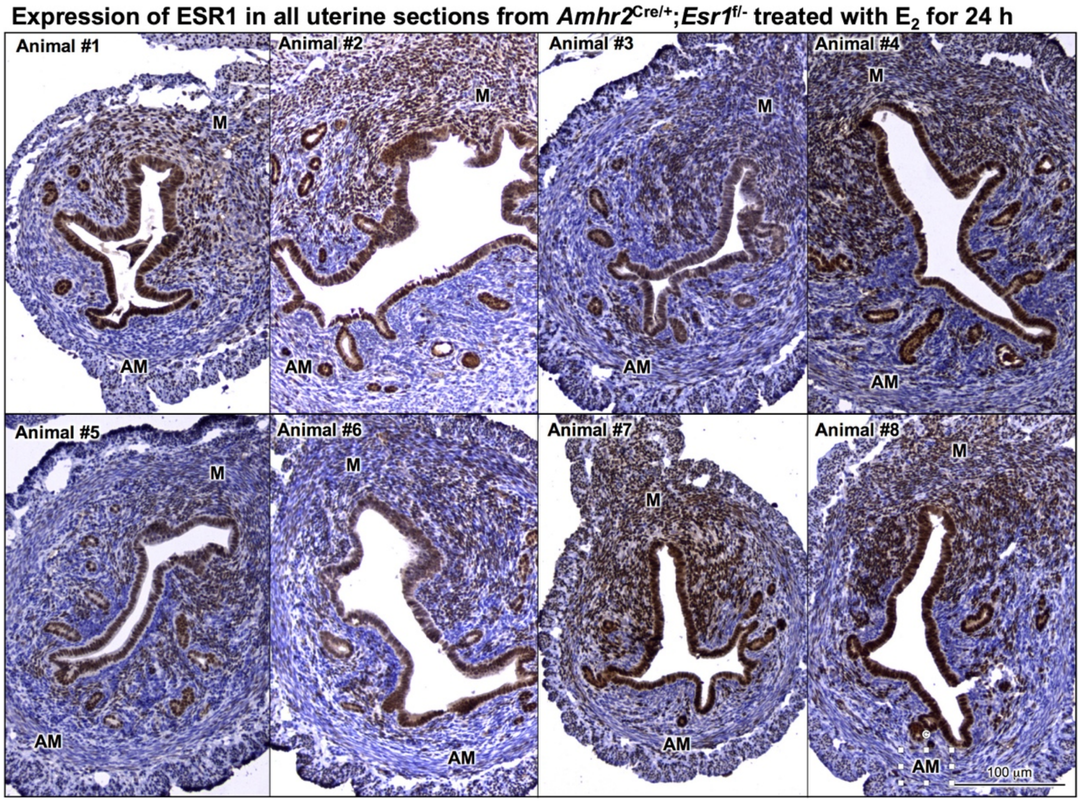

Fig. S1. Expression of ESR1 in *Amhr2*$^{Cre/+}$;*Esr1*$^{f/-}$ animals that were treated with E_2 for 24 h. The images of eight different animals illustrate wide degree of ESR1 deletion in the stromal cell layer and circular muscle cell layer. Each image is oriented with mesometrial poles at the top of the images and anti-mesometrial poles at the bottom of the images. Images were taken at the similar magnification. Scale bar = 100 μm. N=8 animals.

All uterine sections (ESR1 and Ki67 IHC) from *Amhr2*$^{Cre/+}$;*Esr1*$^{f/-}$ treated with E_2 for 24 h

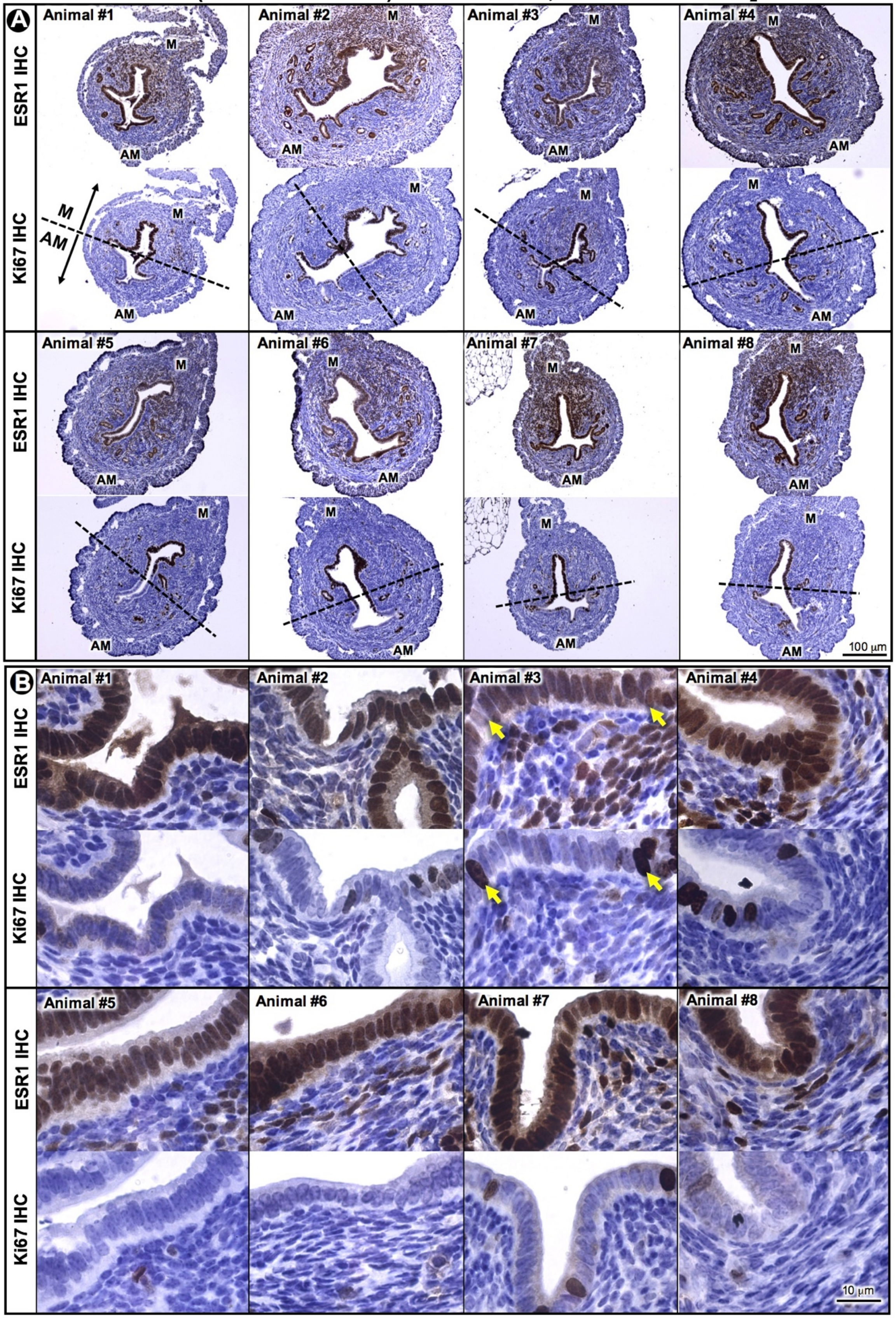

Fig. S2. Expression of ESR1 in comparison to Ki67 in *Amhr2*$^{Cre/+}$;*Esr1*$^{f/-}$ animals that were treated with E_2 for 24 h. (**A**) Whole uterine cross sections were stained with ESR1 and Ki67 antibodies in the adjacent sections of animal #1-8 (order corresponding to **Fig. S1**). Dotted lines indicate the regions of mesometrial (M) vs. anti-mesometrial poles for the calculation of Ki67 positive luminal epithelial cell count (used in Fig. 2*D*). Images were taken at the similar magnification. Scale bar = 100 µm. (**B**) Higher magnification images of ESR1 and Ki67 immunohistochemical staining at the anti-mesometrial poles of Fig S2*A* taken at 100x objective lens. Yellow arrows indicate the expression of ESR1 in the stromal cells adjacent to the luminal epithelial cells, in which proliferating (Ki67-positive cells) are seen. Images were taken at the similar magnification. Scale bar = 10 µm. N=8 animals.

EdU incorporation in the uterine cross-section from stimulated non-responding *Amhr2*$^{Cre/+}$;*Esr1*$^{f/-}$ female uteri

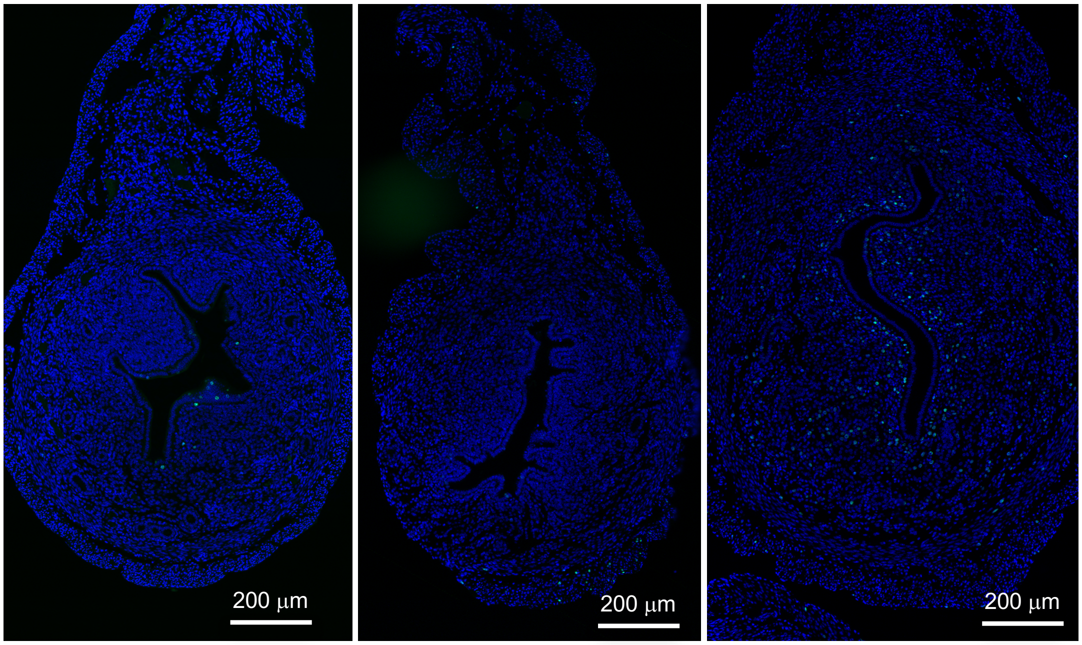

Fig. S3. Representative images of uterine cross-sections from *Amhr2*$^{Cre/+}$;*Esr1*$^{f/-}$ uterine horns that did not respond to artificial decidualization. Green indicates cells in S-phase of DNA synthesis. Blue represents Hoescht stained nuclei.
